# Pharmacy-Inspection Outcomes in Great Britain: An Analysis of Regulatory Compliance and the Impact of Vaccination Service Provision

**DOI:** 10.3390/pharmacy14040107

**Published:** 2026-07-14

**Authors:** Cosmika Goswami, Yoni Carmel, Rosalind Gittins, Gazala Akram

**Affiliations:** 1 Strathclyde Institute of Pharmacy and Biomedical Sciences, University of Strathclyde, Glasgow G4 0RE, UK; cosmika.goswami@strath.ac.uk; 2 General Pharmaceutical Council, London E14 4QJ, UK

**Keywords:** community pharmacy, regulation, inspection outcomes, vaccination, governance

## Abstract

Community pharmacies provide vaccinations through nationally commissioned or private services. In Great Britain, pharmacies are regulated by the General Pharmaceutical Council (GPhC) and inspected for compliance against the 26 Standards for registered pharmacies. GPhC inspection outcomes between 2022 and 2024 were examined for differences between pharmacies offering vaccination services and those who did not. Inspection data from 2197 pharmacies, consisting of 1918 (87.3%) ‘brick-and-mortar’ pharmacies and 279 (12.7%) ‘other’ types (predominantly online pharmacies) were analysed. In total, 1922 (87.5%) pharmacies met all 26 Standards. Pharmacies providing vaccinations demonstrated higher compliance with Standards relating to governance, service delivery, and suitability of premises. Independent single-owner pharmacies and independent pharmacy chains were more likely to not meet standards. A gradient effect was observed across pharmacy-ownership categories, with progressively higher odds of inspection failure as the ownership model size decreased. Analysis of inspector comments identified three key factors influencing inspection performance: governance and organisational oversight; suitability of the physical environment; and staff competence and training. Strengthening governance structures, workforce development and infrastructure support, particularly in smaller independent pharmacies, may improve consistency in regulatory compliance, and support safe, high-quality delivery of vaccination services from community pharmacies.

## 1. Introduction

There has been a notable expansion in the provision of vaccination services by community pharmacies in Great Britain (GB). Their role has progressed from primarily promoting vaccination, increasing public awareness of its benefits, to also administering vaccines: an evolution that became particularly evident during the COVID-19 pandemic [1]. Vaccinations may be provided through commissioned services under the various (predominantly NHS) community pharmacy contracts [2,3] or as a private service, which is more common for travel vaccines [4].

In GB, community pharmacies are regulated by the General Pharmaceutical Council (GPhC), which is “*responsible for assuring that they operate safely and effectively to protect, promote, and maintain the health, safety, and wellbeing of patients and the public*” [5]. The GPhC conducts inspections of pharmacies with a risk-based approach, assessing regulatory compliance with the GPhC Standards for Registered Pharmacies, which define the minimum expectations [6]. The Standards are structured around five key Principles: governance; staff; premises and environment; services; equipment and facilities, which are further subdivided into 26 individual Standards [6] (Table 1).

The Standards provide the regulatory framework through which each pharmacy’s premises are evaluated: the final inspection outcome is reported as either “standards met” or “standards not met”, and enforcement action may be taken where standards are not met. Previous GPhC data insights reports have suggested that pharmacies providing vaccination services may perform better in inspections [7,8]. This may reflect the organisational infrastructure required to deliver vaccination services, including trained staff, appropriate consultation facilities, and governance procedures. However, there has been limited empirical analysis of inspection data to determine whether these differences exist systematically across the sector. This study aims to understand the relationship between pharmacy vaccination services and GPhC inspection outcomes by comparing regulatory compliance between pharmacies offering vaccination services and those that do not.

## 2. Materials and Methods

Published GPhC inspection data from January 2022 to December 2024 was collated and prepared for analysis using Microsoft Excel. This excluded re-inspections (which follow a recently failed inspection) and intelligence-led inspections [9]. This time period was selected as it followed the most significant disruption to pharmacy services and inspections from the COVID-19 pandemic and precedes changes to the inspection model and reports introduced in January 2025 [10]. The dataset contains binary indicators describing compliance with the 26 Standards for Registered Pharmacies, recorded during inspections as either “standard met” or “standard not met”, as well as the inspector’s free-text comments against each standard based upon their observations. Details of clinical services, including the provision of vaccination, were also captured.

Pharmacies were categorised based on ownership identities supplied by pharmacies to the GPhC, although these do not always reflect complex multi-entity ownership structures. Six categories were defined:

- Category 1: Independent single-owner pharmacies (ISO)
- Category 2: Independent pharmacy chains consisting of 2–5 pharmacies (IPC)
- Category 3: Small multiple-pharmacy chains of 6–25 pharmacies (SMPC)
- Category 4: Medium multiple-pharmacy chains of 26–250 pharmacies (MMPC)
- Category 5: Large multiple-pharmacy chains of 251–1000 pharmacies (LMPC)
- Category 6: National pharmacy chains with more than 1000 premises (NPC)

Inspections relating to hospital pharmacies, prison pharmacies, veterinary pharmacies and temporary pharmacy sites (such as festival sites) and dispensing hubs were excluded as these premises typically do not offer vaccinations to the public. The final dataset focused on community pharmacy premises and ‘other’ service models which included internet/distance-selling pharmacies and ‘clinics’ offering aesthetic or travel/holiday services.

Descriptive statistics were used to summarise pharmacy characteristics and inspection outcomes, including compliance rates across the five principles and twenty-six Standards. Compliance between vaccinating and non-vaccinating pharmacies was assessed using a comparative categorical approach, with pass/fail outcomes for each Standard and odds ratios (ORs) calculated to estimate the likelihood of failing a Standard: an OR of 1 indicates no difference between groups, while ORs greater than 1 indicate higher odds of not meeting the standard (i.e., failure) [11].

Differences in compliance across the Standards were evaluated using chi-square tests, with Fisher’s exact test applied when expected cell counts were small (<5). *p*-values were adjusted for multiple comparisons across each standard using the Benjamini–Hochberg method to control false discovery rate [12]. Inspection outcomes (determining compliance with Standards) were additionally examined by pharmacy ownership size. A multivariable logistic regression model (GLM with binomial family) was used to assess the association between vaccination status and inspection outcomes, adjusting for ownership type. Data preparation, statistical code, and outputs were independently reviewed by members of the research team to verify accuracy and reproducibility of the findings.

The free-text comments could either be taken from a bank of typical feedback comments provided by the GPhC or could be bespoke and specific to a situation. Inspectors’ comments relating to the Standards which showed marked differences in inspection outcomes were subjected to initial visualisation and categorisation using Microsoft Excel Copilot, mirroring the process seen in Large Language Machine-assisted content analysis [13,14]. All AI outputs were subjected to academic oversight and rigorous scrutiny by one of the authors (GA). The subsequent coding of potential themes was through adaptation of Braun and Clark’s Thematic Analysis Framework [15] and performed by one of the authors (GA). The final themes (and quotations used) were manually checked and agreed by all authors.

## 3. Results

A total of 2296 GPhC pharmacy routine inspections were reported between January 2022 and December 2024. Ninety-nine (4.3%) pharmacy premises were excluded because they were either hospital, prison, veterinary or ‘temporary’ pharmacies, e.g., festival sites (n = 83) or exclusively registered as Dispensing Hubs (n = 16), which do not offer vaccines, leaving a final cohort of 2197 pharmacies. Most pharmacies (1918, 87.3%) were traditional ‘bricks-and-mortar’ community pharmacies with the remainder (n = 279, 12.7%) consisting of ‘other’ types of pharmacies such as internet or distance-selling pharmacies which sometimes offer vaccine services. The majority of inspected pharmacies were in England (n = 1876, 81.7%), followed by Scotland (n = 241, 10.5%) and Wales (n = 80, 3.5%) (Table 2).

Overall, 1922 pharmacies (87.5%) met all inspection standards and therefore achieved the outcome of “standards met”. Amongst the pharmacies offering vaccines, 90.9% (1352) met all Standards, compared with 80.4% of pharmacies not offering vaccines. Performance across the five Principles generally showed high levels of compliance, exceeding 85% regardless of whether vaccinations were offered as a service or not. Some differences were observed between the two groups. Pharmacies not offering vaccination services demonstrated lower compliance rates with Principle 1 (Governance) and Principle 4 (Services), at 85.2% and 87.6%, respectively, compared with pharmacies offering vaccination services (94.4% and 94.5%).

**Figure 1:**
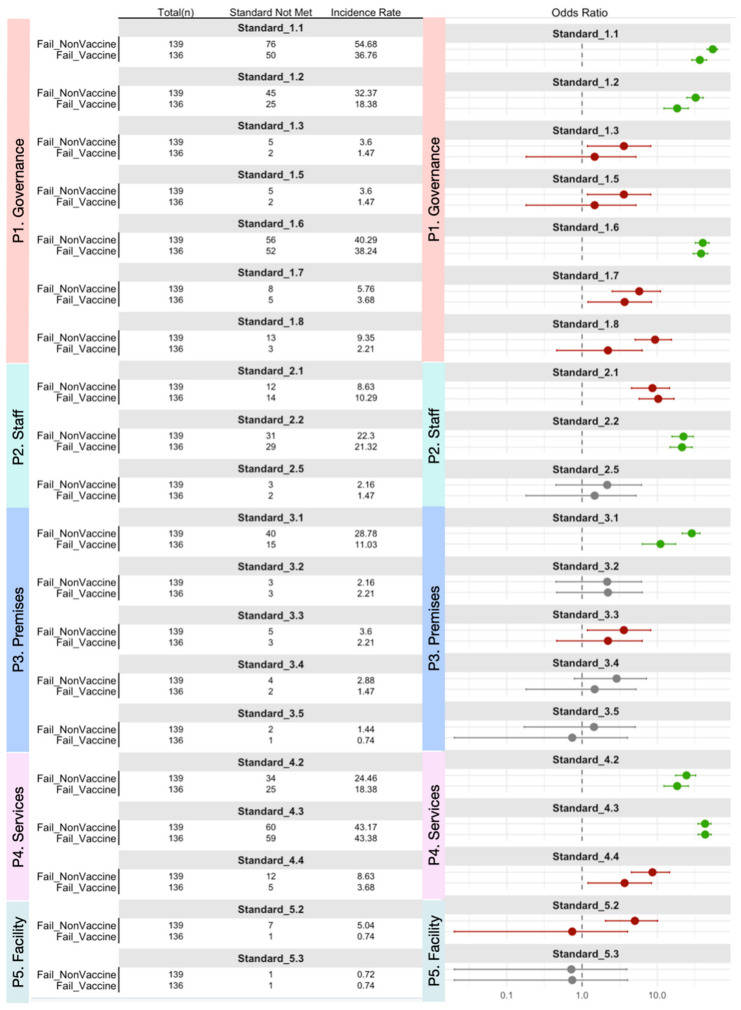
Multivariable logistic regression analysis of inspection failure across vaccination and non-vaccination pharmacies.

Analysis by individual Standards found that seven Standards (Standards 1.1, 1.2, 1.6, 2.2, 3.1, 4.2 and 4.3) had lower levels of compliance, regardless of the vaccination status of the pharmacy (Figure 1).

The Forest plot shows odds ratios (ORs) for failure across 20 Standards, i.e., excluding those Standards with zero counts in both groups. The left-hand columns show, for each Standard, the total number of pharmacies inspected, the number failing that Standard, and the corresponding failure rate. Seven Standards (1.1, 1.2, 1.6, 2.2, 3.1, 4.2 and 4.3), previously identified as having lower compliance, are highlighted in green and show greater deviation from the null value (OR = 1), indicating higher failure rates. (Wider confidence intervals, particularly for Standards 3.5, 5.2 and 5.3, reflect the small number of pharmacies failing these standards and should therefore be interpreted with caution).

Statistical significance of these differences was assessed using Fisher’s Exact Test. The analysis identified statistically significant differences for Standard 1.1 (adjusted *p*-value = 0.05) and Standard 3.1 (*p* < 0.05) at the 95% confidence level: Standard 1.1 relates to risk management and Standard 3.1 relates to suitability of premises.

**Figure 2:**
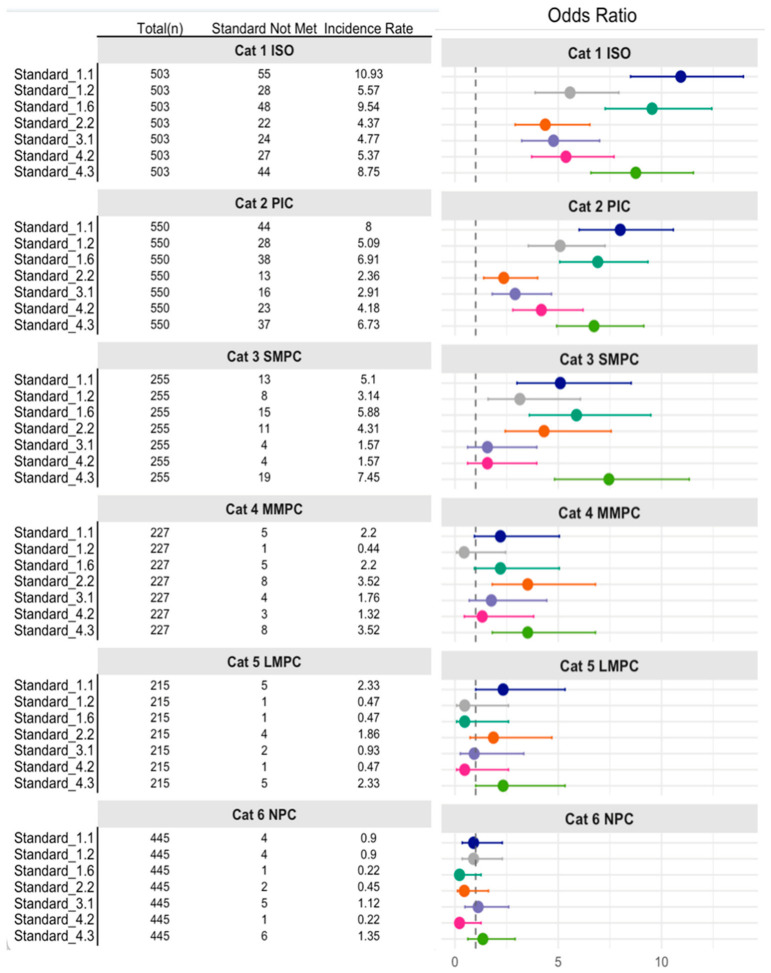
Multivariable logistic regression of inspection failure by pharmacy ownership structure (Categories 1–6). The left hand side columns present the total number of pharmacies, the number of Standards not met, and the corresponding incidence (failure) rates for each Standard.

Inspection outcomes also varied according to the pharmacy ownership structure categories 1–6 (Figure 2). 

To further examine this relationship, odds ratios were calculated. The odds ratio represents how likely pharmacies in each ownership category are to fail a Standard, relative to those where standards are all met. Values greater than 1 indicate higher odds of failing the Sstandard, while values less than 1 indicate lower odds. A logarithmic scale was used to accommodate the wide range of OR values observed across Standards and ownership categories.

A gradient pattern was observed across pharmacy-ownership categories, with progressively higher odds of inspection failure as the size decreased. The corresponding confidence intervals shift further away from the value of 1 (vertical dotted line) for smaller pharmacy-ownership categories, whereas Cat 6 (NPC) showed the lowest risk (estimated OR: ~0.5; 95% CI: 0.1–1.5) and incidence rates falling below 1.5% across all Standards, highlighting a marked improvement in compliance compared to Cat 1 (incidence rates 4–10%). The results showed a consistent trend in which the likelihood of inspection failure increased as the number of premises owned decreased, particularly across the previously identified seven specific standards (1.1, 1.2, 1.6, 2.2, 3.1, 4.2 and 4.3). Multivariate logistic regression identified that pharmacies offering vaccination services were less likely to fail inspection, even after accounting for ownership type (adjusted OR 0.49, 95% CI 0.40–0.64, *p* < 0.005). A sensitivity analysis using ownership size as a quantitative variable produced similar results (adjusted OR 0.50, 95% CI 0.39–0.60), indicating that the association between vaccination-service provision and inspection outcomes was robust to the method used to determine model ownership size.

To further understand the difference in inspection outcomes between pharmacies offering vaccines and those who do not, the comments (N = 99) made by inspectors in relation to the standards with the biggest differences in failure rate [Standards 1.1 (n = 15), 1.2 (n = 15), 3.1 (n = 20) and 4.2 (n = 49)] between the two groups yielded three main themes.

**Theme 1. Inadequate Governance**: This theme reflected ineffective management of dispensing errors or inadequate risk assessments, occurring alongside a general absence of accountability. Additionally, gaps in compliance with standard operating procedures, unresolved actions from previous inspections, and other systemic operation failures which could compromise patient safety were also identified.

“*The pharmacy doesn’t adequately manage all the risks with its services. The pharmacy doesn’t have complete and up-to-date written procedures that reflect the pharmacy’s current practice. This includes the management of near miss errors and dispensing incidents, and the pharmacy’s technology systems. Pharmacy team members are unclear about how to provide services safely in certain circumstances*”

(Health Centre Pharmacy, Cat 1 ISO, NE England)

Internet or distance-selling pharmacies were associated with unique issues relating to their operation, such as the transactional nature of medication requests or misleading website content. The lack of adequate risk assessments by these pharmacies was also particularly evident.

“*The pharmacy does not appropriately review its prescribing service to ensure its processes are effective at keeping people safe*”

(Distance-selling pharmacy, Cat 3 SMPC, Scotland)

**Theme 2. Staffing levels and competency**: Issues associated with poor staffing levels or staff who are lacking in knowledge and skills, often with a lack of access to relevant courses or training, were consistently identified as factors contributing to standards not being met. An undercurrent of a poor safety culture dominated with specific comments about untrained staff or individuals being unclear of their own role, their limitations, and other factors vital to effective pharmacy operations.

“*The pharmacy does not have enough suitably qualified and skilled staff to provide its services safely and effectively. The current staffing arrangements are insufficient to fully cope with the workload, and routine tasks are not being completed or undertaken in a timely manner*”

(High Street Pharmacy, Cat 4 MMPC, SW England)

“*Staff members are undertaking tasks in the absence of the right training*”

(High Street pharmacy, CAT 3 SMPC, W Midlands, England)

Conversely, amongst vaccine-offering pharmacies, there was a strong onus on staff training and development within a wider learning environment.

“*Pharmacy Team members have the appropriate skills, qualifications and competence for their role and the tasks they carry out*”

(High Street Pharmacy, CAT 6 NPC, SE England)

“*The pharmacy is good at supporting its team members to help keep their knowledge and skills up to date. It provides structured ongoing training and protected time to learn while they are at work*”

(High Street Pharmacy, Cat 6 NPC, Wales)

**Theme 3. Physical environment/Fit-for-Purpose premises:** Pharmacies with good ‘housekeeping’ in clean, well laid-out premises or where technology or newer ways of working were adopted, making service provision more effective and/or safer, were more prominent in those offering vaccines.

“*The pharmacy’s new premises have been fitted to a notably high standard of fixtures and fittings. They are of a bespoke design, providing a very professional environment for the services delivered*”

(High Street Pharmacy, CAT 3 SMPC, London England)

“*The pharmacy is well designed to meet the needs of the people who use it*”

(High Street pharmacy, Cat 2 IPC, SE England)

The opposite was generally found in non-vaccine pharmacies where Standards were not met. This included premises with unsuitable medicine storage, or lacking systems for stock rotation, expiry checking, compromised security and/or uncontrolled access to medicines. Pharmaceutical fridges were often found not to work properly or contain inappropriate items, e.g., foodstuff. Medicines with potential to cause harm or be misused were also found not to be suitably managed. In some cases, premises had cleanliness issues.

“*The pharmacy is disorganised and cluttered. It has limited clear space to be able to dispense and check medicines safely. There are items on the dispensary floor which are tripping hazards for staff. And the consultation room is untidy*”

(High Street pharmacy, CAT 1 ISO, London England)

The inadequacy of premises for some internet and distance-selling pharmacies was particularly palpable. There appeared to be limited space for the volume of dispensing, which occurred alongside poor organisation and clutter. This indicated that some premises were not of an appropriate standard required for the provision of pharmacy services.

“*The pharmacy has insufficient storage space for the medicines it has in stock. This is partly due to a lack of experience and understanding about the new pharmacy operating system. The floor is congested with overflow stock and other items. And this creates hazards for team members*”

(Internet pharmacy, Cat 2 IPC NPC, NW England)

“*Pharmacy services are not provided from an environment that is appropriate for the provision of healthcare services. The pharmacy premises do not have enough space to support the pharmacy’s current volume of dispensing*”

(High Street pharmacy, Cat 3 SMPC, SW England)

Overall, pharmacies failing to meet Standards commonly exhibited weak governance and gaps in staff training and supervision, alongside deficiencies in premises and compromised security. In contrast, pharmacies meeting Standards demonstrated stronger governance, structured training, collaborative team environments, and well-organised, appropriately equipped premises. Distance-selling pharmacies were additionally more associated with risks related to website design and inadequate clinical-risk assessment.

## 4. Discussion

This is the first known study to explore GPhC inspection outcomes for pharmacies offering vaccination services. Pharmacies providing vaccination services demonstrated higher levels of compliance across several Standards, particularly those associated with governance, service delivery and premises. The delivery of vaccination services requires particularly robust consideration of the need for more highly trained staff, suitable consultation facilities and clear clinical governance systems as outlined within commissioned services contracts and vaccination-service specifications. Pharmacies that are already experiencing challenges in workforce development, premises suitability, or governance capacity may therefore be less likely to undertake the additional requirements associated with vaccination services. These pharmacies will also not have access to the additional remuneration available through the commissioned services which can be invested in improving the pharmacy. Conversely, pharmacies that have implemented the systems necessary to support vaccination delivery may also exhibit stronger overall compliance with regulatory Standards. This finding demonstrates the value of a supportive commissioning approach which encourages governance maturity and quality improvement activity rather than payment based on volume of activity. Globally, the merits of vaccination services delivered from pharmacy premises are increasingly recognised and are reflected in the various reimbursements systems provided by governments [16].

The inspector’s comments support this association, with pharmacies demonstrating strong inspection performance often characterised by structured governance processes and active monitoring of service quality. Inspectors frequently highlighted evidence of regular review of incidents, documentation of near-miss events, and collaborative team environments that support learning and quality improvement. Such characteristics align with established safety culture frameworks in community pharmacy settings where open communication and continuous improvement are central to maintaining high-quality care [17]. Conversely, pharmacies failing to meet Standards more frequently demonstrated weaknesses in governance systems. These findings are consistent with previous regulatory insight reports [7] which have identified governance and organisational oversight as common areas of non-compliance within community pharmacy practice. Since these inspections were conducted, the GPhC has released updated guidance on providing services at a distance, including online, and ensuring a safe and effective pharmacy team, and subsequently reported an improvement in internet pharmacy performance [8,18,19]. Further research could explore the impact of this guidance.

Pharmacy compliance with regulatory standards can be improved through increased awareness of GPhC Standards and guidance with a particular focus on governance and the continued sharing of data and learnings with the sector [20].

Workforce capability and staff training also emerged as key determinants of inspection performance. Ensuring that pharmacy staff have access to appropriate training and support therefore represents an important component of maintaining safe pharmacy practice. Variation in inspection outcomes was also observed according to pharmacy chain size. Larger organisations may benefit from centralised governance systems, dedicated compliance teams, and centrally standardised operating systems that support consistent regulatory compliance across multiple sites. Smaller independent pharmacies may face greater operational constraints, including limited staffing resources and fewer organisational support structures.

Future research could explore additional organisational factors affecting regulatory compliance, including workload pressures, workforce stability, and organisational culture.

Several limitations should be considered when interpreting the findings of this study. Inspection outcomes represent performance at a specific point in time and do not fully capture the pharmacy’s ongoing practice. Therefore, the relationship between vaccination-service provision and inspection outcomes should not be interpreted as causal. Furthermore, this analysis does not differentiate between different vaccination-service models, as there may be variation between NHS or other locally commissioned or private services: this could be explored in future research.

Despite these limitations, the study provides valuable insight into patterns of regulatory compliance across GB community pharmacy practice. By combining inspection outcomes with inspector observations, a deeper understanding of organisational factors influencing inspection performance is provided. Longitudinal studies examining changes in inspection outcomes over time may also help identify how pharmacies respond to regulatory feedback and quality improvement initiatives.

## 5. Conclusions

GPhC-registered community pharmacies across GB generally demonstrate high levels of regulatory compliance at inspection. However, pharmacies providing vaccination services appear to demonstrate stronger compliance across several Standards, particularly those related to governance, staffing and premises suitability. Supportive commissioning approaches, increased awareness of GPhC Standards and guidance documents will lead to improved compliance in the sector. As community pharmacies continue to expand their role in delivering vaccination services, maintaining robust regulatory standards will remain essential for ensuring patient safety and public trust.

## Figures and Tables

**Table 1:** GPhC Pharmacy Premises Principles and Standards for Inspection.

Principle	Standard
1. The governance arrangements safeguard the health, safety and wellbeing of patients and the public	1.1. The risks associated with providing pharmacy services are identified and managed
	1.2. The safety and quality of pharmacy services are reviewed and monitored
	1.3. Pharmacy services are provided by staff with clearly defined roles and clear lines of accountability.
	1.4. Feedback and concerns about the pharmacy, services and staff can be raised by individuals and organisations, and these are taken into account and action taken where appropriate.
	1.5. Appropriate indemnity or insurance arrangements are in place.
	1.6. All necessary records for the safe provision of pharmacy services are kept and maintained.
	1.7. Information is managed to protect the privacy, dignity and confidentiality of patients and the public who receive pharmacy services.
	1.8. Children and vulnerable adults are safeguarded.
2. Staff are empowered and competent to safeguard the health, safety and wellbeing of patients and the public	2.1. There are enough staff, suitably qualified and skilled, for the safe and effective provision of the pharmacy services provided. 2.2. Staff have the appropriate skills, qualifications and competence for their role and the tasks they carry out, or are working under the supervision of another person while they are in training. 2.3. Staff can comply with their own professional and legal obligations and are empowered to exercise their professional judgement in the interests of patients and the public. 2.4. There is a culture of openness, honesty and learning. 2.5. Staff are empowered to provide feedback and raise concerns about meeting these standards and other aspects of pharmacy services. 2.6. Incentives or targets do not compromise the health, safety or wellbeing of patients and the public, or the professional judgement of staff.
3. The environment and condition of the premises from which pharmacy services are provided, and any associated premises, safeguard the health, safety and wellbeing of patients and the public	3.1. Premises are safe, clean, properly maintained and suitable for the pharmacy services provided. 3.2. Premises protect the privacy, dignity and confidentiality of patients and the public who receive pharmacy services. 3.3. Premises are maintained to a level of hygiene appropriate to the pharmacy services provided. 3.4. Premises are secure and safeguarded from unauthorised access. 3.5. Pharmacy services are provided in an environment that is appropriate for the provision of healthcare.
4. The way in which pharmacy services, including the management of medicines and medical devices, are delivered safeguards the health, safety and wellbeing of patients and the public	4.1. The pharmacy services provided are accessible to patients and the public 4.2. Pharmacy services are managed and delivered safely and effectively 4.3. Medicines and medical devices are: • obtained from a reputable source • safe and fit for purpose • stored securely • safeguarded from unauthorised access • supplied to the patient safely • disposed of safely and securely 4.4. Concerns are raised when it is suspected that medicines or medical devices are not fit for purpose.
5. The equipment and facilities used in the provision of pharmacy services safeguard the health, safety and wellbeing of patients and the public.	5.1. Equipment and facilities needed to provide pharmacy services are readily available 5.2. Equipment and facilities are: • obtained from a reputable source • safe to use and fit for purpose • stored securely • safeguarded from unauthorised access • appropriately maintained 5.3. Equipment and facilities are used in a way that protects the privacy and dignity of the patients and the public who receive pharmacy services.

**Table 2:** Demographics of inspected community pharmacies.

	Total N = 2197	Vaccinating Pharmacies N = 1488 (67.7%)	Non-Vaccinating Pharmacies N = 709 (31.3%)
**Pharmacy Type**			
Traditional Comm Pharm	1918 (87.3%)	1436 (96.5%)	482 (67.9%)
Other	279 (12.7%)	52 (3.5%)	227 (32.1%)
**Location**			
England	1876 (85.3%)	1313 (88.2%)	563 (79.4%)
Scotland	241 (10.9%)	104 (6.9%)	137 (19.3%)
Wales	80 (3.6%)	71 (4.7%)	9 (1.3%)
Ownership size *			
Cat 1 ISO	503 (22.9%)	275 (18.5%)	228 (32.1%)
Cat 2 IPC	550 (25.0%)	343 (23.0%)	207 (29.2%)
Cat 3 SMPC	255 (11.6%)	175 (11.7%)	80 (11.2%)
Cat 4 MMPC	227 (10.3%)	174 (11.7%)	53 (7.5%)
Cat 5 LMPC	215 (9.8%)	173 (11.6%)	42 (5.9%)
Cat 6 NPC	445 (20.2%)	346 (23.2%)	99 (13.9%)
**Inspection Outcome**			
Standards all met	1922 (87.5%)	1352 (90.9%)	570 (80.4%)
Standards not all met	275 (12.5%)	136 (9.1%)	139 (19.6%)
**Performance against Principles 1–5**			
P1 Standards met	2009 (91.4%)	1405 (94.4%)	604 (85.2%)
P1 Standards not all met	188 (8.6%)	83 (5.6%)	105 (14.8%)
P2 Standards met	2113 (96.2%)	1445 (97.1%)	668 (94.3%)
P2 Standards not all met	84 (3.8%)	43 (2.8%)	41 (5.7%)
P3 Standards met	2124 (96.7%)	1466 (98.5%)	658 (92.8%)
P3 Standards not all met	73 (3.3%)	22 (1.5%)	51 (7.2%)
P4 Standards met	2027 (92.2%)	1406 (94.5%)	621(87.6%)
P4 Standards not all met	170 (7.7%)	82 (5.5%)	88 (12.4%)
P5 Standards met	2185 (99.4%)	1486 (99.9%)	699 (98.6%)
P5 Standards not all met	11 (0.6%)	2 (0.1%)	10 (1.4%)

* Two pharmacies missing ownership data.

## Data Availability

The original contributions presented in this study are included in the article. Further inquiries can be directed to the corresponding author.
